# Small-Conductance Ca^2+^-Activated Potassium Type 2 Channels Regulate the Formation of Contextual Fear Memory

**DOI:** 10.1371/journal.pone.0127264

**Published:** 2015-05-04

**Authors:** Saravana R. K. Murthy, Tessi Sherrin, Chad Jansen, Ingrid Nijholt, Michael Robles, Amalia M. Dolga, Nicolas Andreotti, Jean-Marc Sabatier, Hans-Guenther Knaus, Reinhold Penner, Cedomir Todorovic, Thomas Blank

**Affiliations:** 1 Section on Cellular Neurobiology, Program on Developmental Neuroscience, Eunice Kennedy Shriver National Institute of Child Health and Human Development, Bethesda, Maryland, United States of America; 2 Department of Cell & Molecular Biology, John A. Burns School of Medicine, University of Hawaii, Honolulu, Hawaii, United States of America; 3 Laboratory of Cell and Molecular Signaling, The Queen’s Medical Center, Honolulu, Hawaii, United States of America; 4 Isala Academy, Isala Clinic, Zwolle, The Netherlands; 5 Department of Pharmacology and Toxicology, Philipps-University Marburg, Marburg, Germany; 6 Laboratoire INSERM UMR1097, Parc scientifique et technologique de Luminy, Marseille, cedex 09, France; 7 Division for Molecular and Cellular Pharmacology, Medical University Innsbruck, Innsbruck, Austria; 8 Institute for Neuropathology, University of Freiburg, Freiburg, Germany; University of Alabama at Birmingham, UNITED STATES

## Abstract

Small-conductance, Ca^2+^ activated K^+^ channels (SK channels) are expressed at high levels in brain regions responsible for learning and memory. In the current study we characterized the contribution of SK2 channels to synaptic plasticity and to different phases of hippocampal memory formation. Selective SK2 antisense-treatment facilitated basal synaptic transmission and theta-burst induced LTP in hippocampal brain slices. Using the selective SK2 antagonist Lei-Dab^7^ or SK2 antisense probes, we found that hippocampal SK2 channels are critical during two different time windows: 1) blockade of SK2 channels before the training impaired fear memory, whereas, 2) blockade of SK2 channels immediately after the training enhanced contextual fear memory. We provided the evidence that the post-training cleavage of the SK2 channels was responsible for the observed bidirectional effect of SK2 channel blockade on memory consolidation. Thus, Lei-Dab^7^-injection before training impaired the C-terminal cleavage of SK2 channels, while Lei-Dab^7^ given immediately after training facilitated the C-terminal cleavage. Application of the synthetic peptide comprising a leucine-zipper domain of the C-terminal fragment to Jurkat cells impaired SK2 channel-mediated currents, indicating that the endogenously cleaved fragment might exert its effects on memory formation by blocking SK2 channel-mediated currents. Our present findings suggest that SK2 channel proteins contribute to synaptic plasticity and memory not only as ion channels but also by additionally generating a SK2 C-terminal fragment, involved in both processes. The modulation of fear memory by down-regulating SK2 C-terminal cleavage might have applicability in the treatment of anxiety disorders in which fear conditioning is enhanced.

## Introduction

Apamin-sensitive, small-conductance Ca^2+^-activated K^+^ channels (SK channels 1–3) modulate neuronal excitability of hippocampal neurons. The mouse hippocampal formation displays high expression levels of SK1 and SK2 proteins and moderate levels of SK3 channel protein [1]. Apamin block of SK channel activity was shown to enhance hippocampal synaptic plasticity induced by high-frequency stimulation [2] and to accelerate hippocampus-dependent non-aversive spatial and contextual fear memory encoding [2,3]. Although apamin has some preference for SK2 channels, it also affects other subtypes of the SK channel family [4,5]. In contrast to the effects of blocking SK channel activity, increase in SK channel activity impairs learning. For example, systemically applied SK channel agonists 1-EBIO and CyPPA impair the encoding of object memory in a spontaneous object recognition task [3], while injection of the SK channel activator NS309 in the hippocampal CA1 region slows the acquisition rate and magnitude of the hippocampus-dependent trace eyeblink conditioning task [6].

With the development of novel genetic mouse models, it has become possible to specifically focus on SK2 channels and to identify the contribution of this SK channel subtype to synaptic plasticity and memory formation. In transgenic mice that overexpress SK2 channels by 10 fold, both hippocampal learning and memory and synaptic plasticity in hippocampal slices were impaired [7]. From the finding that the contextual fear memory impairment was eliminated when mice were permitted longer pre-exposure to the conditioning chamber, it was speculated that SK2 channels restrict the encoding and not the retention or retrieval of hippocampal fear memory [8]. However, hippocampal SK2 channels seem to contribute only to certain aspects of hippocampal cognitive functions as indicated by the finding that SK2 channel blockade had no effect in an olfactory associative task [9]. Apparently, other SK channel subtypes have to be involved in this behavioral task because apamin facilitated consolidation of new odor associations [9]. In addition, the use of SK2 overexpressing or SK2 deficient mice does not allow to determine the potential role of SK2 channels in the different phases of fear memory formation. Thus, reversible pre- and post-training manipulations offer advantages over permanent, genetic approaches in order to characterize the involvement of SK2 channels in acquisition, consolidation and retention of contextual fear. In the current study, we investigated the specific involvement of the SK2 channel subtype in hippocampal synaptic plasticity and in different phases of hippocampus-dependent contextual fear conditioning using selective antisense probes against SK2 and a highly selective antagonist for SK2, Lei-Dab^7^ [10].

## Materials and Methods

### Animals and Ethics Statement

Experiments were carried out on male C57BL/6J mice (Jackson Laboratories) aged 9–12 weeks. The Institutional Animal Care and Use Committee of the University of Hawaii approved all studies using animals (IACUC: 09-863-6). Animals are provided with care and healthy conditions during their stay in the facility.

### Cannulation

Double guide cannulae were implanted using a stereotactic holder as previously described [11,12]. Double guide cannulae (C235, Plastics One, Roanoke, VA) were implanted using a stereotactic holder during 1.2% avertin anesthesia. The cannulae were placed into both lateral brain ventricles, with anteroposterior (AP) coordinates zeroed at Bregma AP 0 mm, lateral 1 mm, depth 3 mm or directed toward both dorsal hippocampi, AP -1.5 mm, lateral 1 mm, depth 2 mm. For intra-cortical injections, cannulae were placed at AP -1.5 mm, lateral 1 mm and 1.5 mm depth. The animals were allowed to recover for 4–5 d before the experiments started. On the day of the experiment, bilateral injections were performed using an infusion pump (CMA/Microdialysis) at a constant rate of 0.5 or 0.33 ml/min. Upon completion of the experiments, mice were anesthetized with isoflurane, infused with 0.2 μl methylene blue and transcardially perfused with 0.9% saline followed by 4% formalin. Following extraction from the skull, brains were removed, immersed in 4% formalin for 48 h and then transferred into 30% sucrose—formalin solution. Serial 50 μm brain sections were cut, and methylene blue staining was analyzed to locate the positions of the cannulae tip site. For electrophysiological experiments double cannula placement was verified by unilateral methylene blue injection.

### Fear Conditioning

The fear conditioning experiments were performed as previously described [11,13] using a computer-controlled fear conditioning system (TSE, Bad Homburg, Germany). The training conditioning consisted of a single trial. The mouse was exposed to the conditioning context (180 sec) followed by a tone (30 sec) serving as conditional stimulus. During the last 2 sec of the tone, a foot-shock (0.5 or 0.7 mA) serving as unconditional stimulus was delivered through a stainless steel grid floor. Different animals were used for the 0.5 and 0.7 mA foot-shock. Mice remained in the fear conditioning box for 3 min following the foot-shock in order to determine post-shock freezing. In some experiments mice were preexposed to the training context for 5 min. In context preexposure effect experiment the mice were trained using a 20 sec placement to shock interval 24 h later. Animals were placed in the training context and received a single shock (0.5 mA) 20 sec later. Conditioned freezing was assessed as the behavioral parameter of the learned reaction of mice by a time-sampling procedure every 10 s throughout memory tests. Memory tests were performed 24 h after fear conditioning procedures.

### Hippocampal Slice Electrophysiology

Electrophysiological experiments were performed as described previously [11]. Briefly, mice were anesthetized with 2.5% Avertin. After decapitation, hippocampi (8–12 weeks) were rapidly removed and briefly chilled in ice-cold artificial CSF (ACSF) consisting of the following: 130 mM NaCl, 3.5 mM KCl, 10 mM glucose, 1.25 mM NaH_2_PO_4_, 2.0 mM CaCl_2_, 1.5 mM MgSO_4_ and 24 mM NaHCO_3_ (equilibrated with 95% O_2_/5% CO_2_, pH 7.4). Transverse slices 400 μm thick were prepared with a Vibratome (Leica; VT1200S) and maintained at least 1 h in a holding chamber containing aCSF. Extracellular field potentials were recorded in a recording chamber maintained at 32°C. All recordings were made using a SEC-05L amplifier (npi Electronics, Tamm, Germany). Field EPSPs (fEPSPs) were recorded from the stratum radiatum of CA1 in response to stimulation of the Schaffer collaterals stimulated with a bipolar electrode placed on the surface of the slice. Current test intensity was 50–60% of the maximum fEPSP. fEPSPs were measured by taking the slope of the rising phase between 5% and 60% of the peak response. LTP was induced by theta burst stimulation (TBS), at the test pulse intensity, consisting of 5 x 100 Hz bursts with a 200 ms interburst interval and measured 50–60 min after stimulation. Traces were stored on a computer using Pulse 7.4 software for off-line analysis (HEKA, Lambrecht, Germany).

### Jurkat cells Electrophysiology

Whole-cell Patch-clamp experiments were performed at room temperature (20–25°C). Whole-cell currents were recorded by EPC-9 (HEKA, Bellmore, NY) and Patchmaster v2.4 (HEKA). The voltages were corrected for a liquid junction potential of 10 mV. The patch pipettes were pulled from borosilicate glass with resistances of 2.5–3.5 MΩ when the pipettes were filled with internal solution. Cells were held at 0 mV holding potential and stimulated with voltage ramps of 100 ms duration from -100 mV to 100 mV delivered at 0.5 Hz. The currents were filtered at 2.9 kHz and digitized at 10 kHz. Current-voltage (I-V) relationships were obtained from high-resolution ramp currents and the time course of current development was derived from the individual ramp currents by measuring the current corresponding to the ramp voltage of 80 mV plotted over experimental time. Time course data were normalized to cell size as current density (pA/pF) and displayed as mean ± standard error of mean (s.e.m.). Jurkat cells were cultured at 37°C with 5% CO_2_ in RPMI 1640 supplemented with 10% fetal bovine serum (FBS). Jurkat cells were plated on polylysine coated coverslips and bathed in an external solution that contained (in mM) 140 NaCl, 2.8 KCl, 2 MgCl_2_, 10 HEPES, 1 CaCl_2_, 10 glucose, pH 7.3 adjusted with NaOH. The internal solution contained (in mM) 140 K Glutamate, 8 NaCl, 1 MgCl_2_, 10 HEPES, 5 CaCl_2_, 10 Dibromo-BAPTA, pH 7.2 adjusted with KOH. Free Ca^2+^ concentration of the internal solution was 1.9 μM as calculated by WebMaxC (http://web.stanford.edu/~cpatton/webmaxcS.htm). The SK2-LZ peptide was added to the external solution at 100 μM and applied to the cells by a wide-tipped puffer pipette.

### Peptide synthesis of the SK2 Leucine Zipper (LZ) Domain

Mouse SK2-LZ (ISDLNERSEDFEKRIVTLETKLETLIGSIHAL) and a random control peptide (GRSSDEEEDRLAITEIIFNSILEKVTTKLLHL) were synthesized with an ABI 433A peptide synthesizer (Applied Biosystems, Foster City, CA, USA). Couplings were carried out using TentaGel S RAM Resin (90 um), Rink-type Resin (Peptides International, USA) on Fmoc-amino acids cartridges (Applied Biosystems, Foster City, CA, USA) and HBTU (O-Benzotriazole-N, N, N', N '-tetramethyl-uronium-hexafluoro-phosphate (GL Biochem, Shanghai). Peptides were cleaved from the resin with a cleaving mixture containing 0.75 g phenol, 0.25 ml 1,2 ethanedithiol, 0.5 ml thioanisole, 0.5 ml water and 10 ml trifluoroacetic acid and then purified by reversed-phase HPLC on a semipreparative Vydac C-18 column. The purified product was characterized by MALDI-TOF mass spectrometry on a PerSeptive Biosystems Voyager Mass Spectrometer.

### Immunoblot

For biochemical assays, mice were sacrificed by cervical dislocation, hippocampi were dissected out and immediately homogenized at 4°C with a plastic homogenizer in a homogenization buffer containing 50 mM Tris-HCl (pH 8.0), 10 mM EDTA, 4 mM EGTA, 15 mM sodium phosphate, 100 mM β-glycerophosphate, 10 mM sodium fluoride and a protease inhibitor cocktail tablet (Boehringer Mannheim, Germany). Equal amounts of protein for each group were separated on a 12% SDS gel and transferred to an Immobilon-P membrane (Millipore Corporation, Bedford, MA, USA). Blots were probed using crude serum of anti-SK2_(538–555)_ at a dilution of 1:5000 or with affinity-purified anti- SK1_(12–29)_, 1 ng/μl IgG or affinity-purified anti-SK3_(504–522)_, 2 ng/μl IgG [1]. Blots were stripped and then re-probed with GAPDH (1:2500, Abcam, Cambridge, MA) for normalization. Western blots were developed using the chemiluminescence method. Bands were quantified by densitometry (WinCam 2.2 Cybertech).

### RT-PCR

Semi-quantitative reverse transcriptase polymerase chain reaction (RT-PCR) was performed to quantify SK2 transcripts using SUPERSCRIPT One-step RT-PCR with PLATINUM *Tag* (Invitrogen, CA, USA). Hypoxanthine phosphoribosyl transferase (HPRT) was used as housekeeping gene. Primer sequences were SK2-f; 5’-tccgacttaaatgaaaggag-3’, SK2-r; 5’-gctcagcattgtaggtgac-3’, HPRT-f; 5’-cctgctggattacattaaagcactg-3’ and HPRT-r; 5’-cctgaagtactcattatagtcaagg-3’. 1.5 μg of RNA was used for each RT-PCR reaction. Amplified PCR products were separated on 1.5% agarose gels with Tris-borate EDTA buffer and stained with ethidium bromide. Gels were captured as a digital image and quantified by densitometry (WinCam 2.2, Cybertech).

### Statistics

Statistical comparisons were made by using Student’s *t*-test, and one- or two-way ANOVA, followed by Bonferroni correction for multiple comparisons. Data were expressed as mean ± SEM. Significance was determined at the level of *p* < 0.05.

## Results

We used both, a pharmacological and an antisense knock-down approach, to determine the impact of SK2 channels on TBS-induced LTP in the hippocampus and the acquisition, consolidation and retrieval of contextual fear memory.

### Downregulation of Hippocampal SK2 Channels by Specific Antisense Oligonucleotides

**Fig 1:**
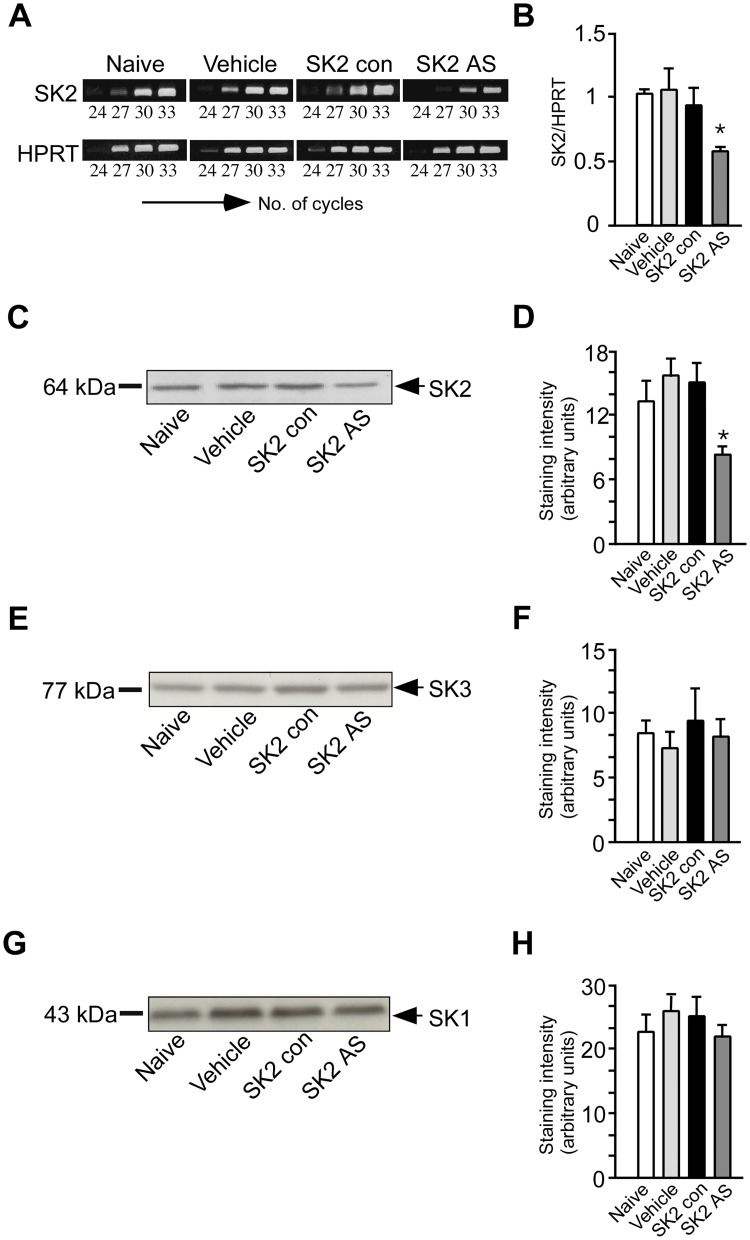
Antisense-SK2 treatment reduced expression of hippocampal SK2 channels. (A) RT-PCR analysis was performed on RNA extracted from a single hippocampus of naïve mice or from a single hippocampus of mice that were pre-treated with vehicle, control oligonucleotides (ODNs) or SK2 antisense ODNs. Each reaction mixture contained a set of primers specific for the cDNA of hypoxantine-phosphoribosyl-transferase (HPRT), used as internal control. (B) Bar graphs show the relative band intensities on the basis of densitometric analysis as ratios of SK2 and HPRT mRNA after 27 cycles of co-amplification from 7–8 mRNA samples. Representative western blots showing the analysis of SK2 (C), SK3 (D) and SK1 (G) protein homogenates from single hippocampi of mice pre-treated with vehicle, control oligonucleotides (ODNs) or SK2 antisense ODNs. Bars represent mean western blot band intensities ± SEM for SK2 (D), SK3 (F) and SK1 (H) proteins from hippocampal homogenates (n = 9) (Bonferroni multiple comparisons test: * *p* < 0.05).

First, we tested the effectiveness of an antisense oligonucleotide (ODN) probe designed to target SK2 channels. Hippocampal tissue was collected 2 h after intracerebroventricular (i.c.v.) ODN or control injection and assayed for the SK2 channel transcript (Fig 1A and 1B) and its encoded protein (Fig 1C and 1D). Out of the investigated time points (2h, 4h, 6h and 8h) the injection time point of 2 h was chosen because it demonstrated the most pronounced effect (data not shown). Both, SK2 channel transcript (ANOVA, F_3, 28_ = 3.7; *p* < 0.05) (Bonferroni correction, *p* < 0.05; SK2 AS vs. Naive) (Fig 1A and 1B) and protein (ANOVA, F_3, 32_ = 4.6; *p* < 0.05) (Bonferroni correction, *p* < 0.05; SK2 AS vs. Naive) (Fig 1C and 1D) were significantly reduced after injection of SK2 channel antisense ODNs, whereas no effect on the amount of SK2 channel transcript (ANOVA, F_3, 28_ = 1.2; *p* > 0.05) and protein (ANOVA, F_3, 28_ = 1.1; *p* > 0.05.) was observed after treatment with vehicle or control ODNs when compared to untreated hippocampi. None of the treatments had any significant effect on hippocampal SK3 or SK1 protein levels (Fig 1E–1H).

### Hippocampal Basal Synaptic Transmission and fEPSP-LTP after Downregulation of SK2 Channels

**Fig 2:**
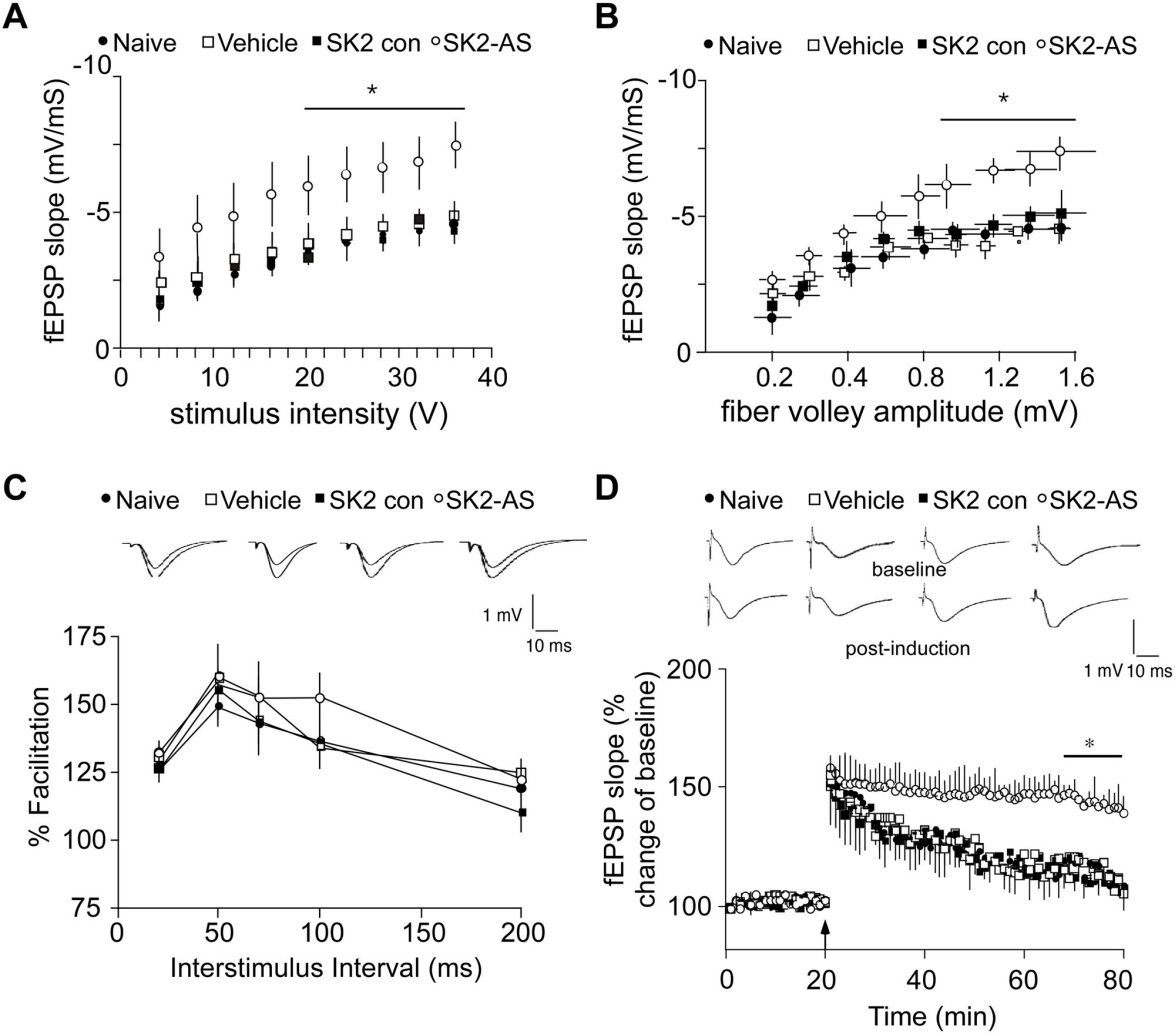
Enhanced basal synaptic transmission and LTP in hippocampal slices from mice pre-treated with SK2 antisense ODNs. The distance between the stimulating and recording electrodes was kept constant between slices. (A) Input-output curve of fEPSP slope (mV/ms) versus stimulus (V) at the SC-CA1 pyramidal cell synapse in naive mice and mice pre-treated with vehicle, antisense ODNs against SK2 channels and control ODNs. The maximal fEPSP slopes were significantly larger in the SK2 antisense-treated mice than those in the naive, vehicle and control ODNs-treated mice. Data are presented as mean ± s.e.m. (B) Relationship between the slope of the evoked fEPSPs from panel A and the corresponding fiber volley amplitude. SK2 antisense-treated mice exhibit a greater postsynaptic response than control groups to similar presynaptic depolarization. Data are presented as mean ± s.e.m. (C) Comparison of PPF in naive mice and mice pre-treated with vehicle, antisense ODNs against SK2 channels and control ODNs. No differences were found between these four groups of mice. Data presented are the mean ± SEM of the facilitation of the second response relative to the first response. Insets: Field EPSPs recorded in response to paired-pulse stimulation at an interstimulus interval of 50 ms in slices from all four treatment groups as indicated. (D) TBS-LTP elicited in slices from mice that were pre-treated with SK2 antisense ODNs was significantly enhanced when compared to LTP induced in slices from naive mice. There was no statistical difference between control ODNs-injected mice and mice that were pre-injected with vehicle. Insets: Responses shown are fEPSPs recorded during baseline (upper row) and 55–60 min (bottom row) after the induction of LTP (post-induction). Traces are averages of five consecutive responses. Statistical significance was determined by two-way ANOVA followed by Bonferroni multiple comparisons test (*p < 0.05).

The effect of SK2 channel downregulation on the properties of synaptic transmission and plasticity was studied by conducting field recordings in the CA1 region of the hippocampus. In evaluating the input-output curve for synaptic function, we found significant treatment-dependent differences in measures of baseline synaptic transmission. For a range of intensities, the mean slope of fEPSPs in the SK2 channel antisense-treated mice (8 slices, 8 mice; Bonferroni correction, *p* < 0.05) were significantly larger in the higher range of stimulus intensities than mean fEPSPs slopes from naïve (11 slices, 7 mice), vehicle-injected (12 slices, 8 mice) and control ODNs-injected (15 slices, 7 mice) mice (two way repeated-measures ANOVA for stimulus and treatment, main effect of treatment: F_3, 574_ = 3.4; *p* < 0.05) (Bonferroni correction, *p* < 0.05; SK2 AS vs. Naïve mice) (Fig 2A). Likewise, when the fEPSPs slopes were plotted as a function of fiber volley slopes, the SK2 channel antisense-treated mice showed enhanced fEPSPs in response to the increasing afferent stimulation (two way repeated-measures ANOVA for stimulus and treatment, main effect of treatment: F_3, 521_ = 5.4; *p* < 0.05) (Bonferroni correction, *p* < 0.05; SK2 AS vs. Naïve mice) (Fig 2B). Subsequently, we analyzed paired-pulse facilitation to test whether presynaptic function was altered. No differences in the degree of facilitation between all four groups were found (two way repeated-measures ANOVA for interval and treatment, main effect of treatment: F_3, 252_ = .39; *p* > 0.05) (Fig 2B). Thus, it is not likely that the enhancement in basal synaptic responses in the SK2 channel antisense-treated mice is caused by presynaptic changes. Since SK2 channels contribute to LTP at Schaffer collateral-CA1 synapses [7,14] one would expect that LTP is affected by downregulation of SK2 channels. We tested this possibility by LTP recordings in the CA1 region of the hippocampus. Theta-burst stimulation (TBS) induced only a weak, descresent form of fEPSP-LTP in hippocampal slices from naïve animals (118 ± 5% of baseline; 11 slices, 7 mice) and from animals pre-injected with vehicle (114 ± 6%; 12 slices, 8 mice) or SK2 channel control-ODNs (115 ± 4%; 15 slices, 7 mice). Only in hippocampal slices prepared from animals i.c.v. injected with antisense ODNs against SK2 channels 2 h before decapitation, weak TBS resulted in significantly enhanced fEPSP-LTP (143 ± 4% of baseline; 8 slices, 8 mice) (F_3, 42_ = 6.9; *p* < 0.05, ANOVA with repeated-measures, last 10 min) (Bonferroni correction, *p* < 0.05; SK2 AS vs. Naïve mice) (Fig 2C).

### Effect of SK2 Channel Inhibition on Contextual Fear Conditioning

**Fig 3:**
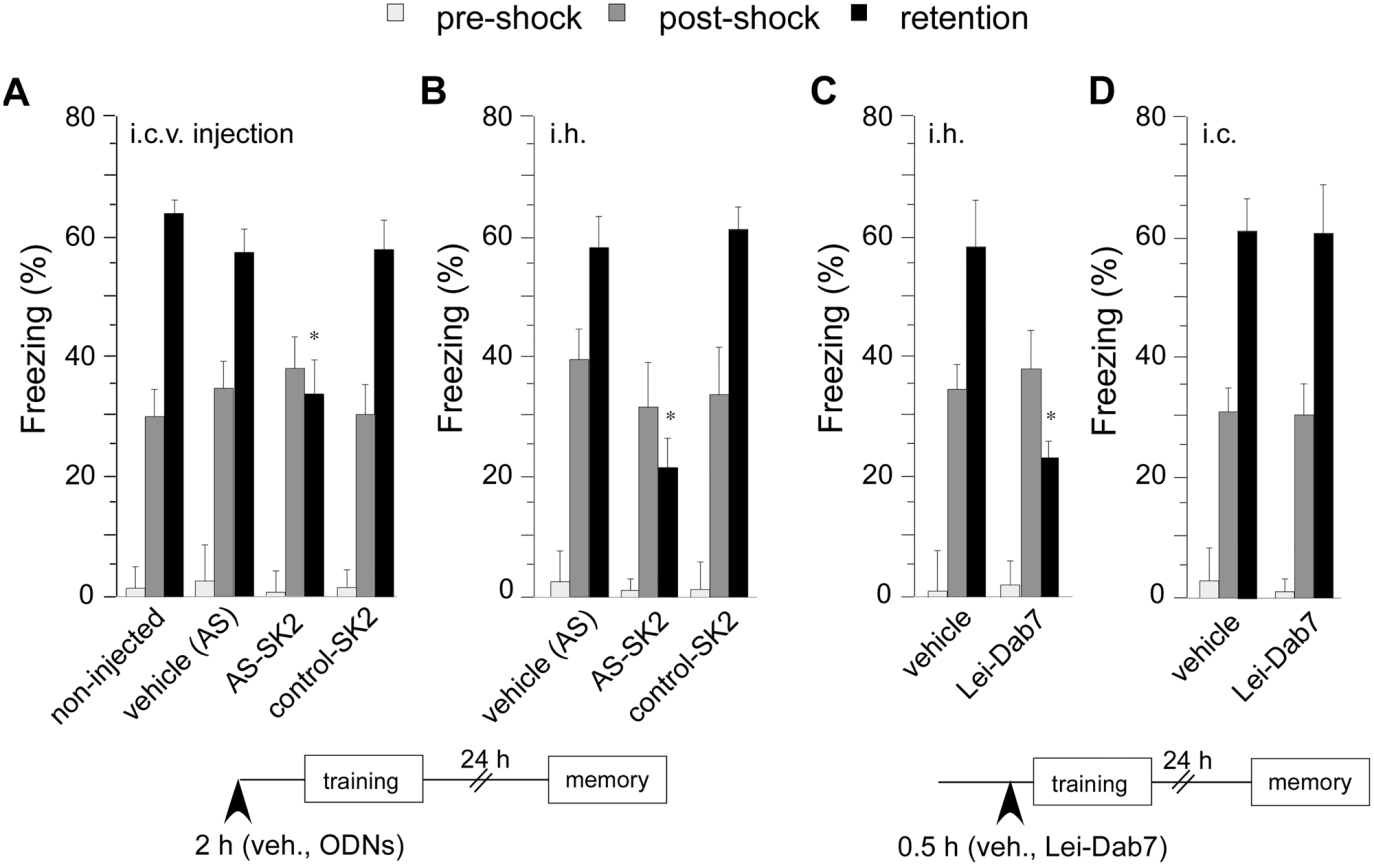
Inhibition of SK2 channel function impaired contextual fear memory. (A, B) Mice injected intrahippocampally (i.h.) or intracerebroventricularly (i.c.v.) with antisense-SK2 ODNs showed reduced freezing when compared to naive animals. No significant difference in freezing scores was seen in mice injected with vehicle or control ODNs (n = 9–11/ group). (C) Intrahippocampal injection of the selective SK2 antagonist Lei-Dab^7^ impaired freezing when compared to non-injected animals, whereas vehicle-injection had no effect (n = 8–9/group). (D) When Lei-Dab^7^ was injected intracortically (i.c.), freezing was not different from non-injected animals. Similarly, vehicle-injected mice showed no significant difference in freezing when compared to non-injected mice (n = 6/ group). Percentage of freezing during pre-shock and post-shock was not significantly different in any treatment group from percentage of freezing of non-injected animals. Freezing was measured in the memory test 24 h after training. Data presented are the mean ± SEM. Statistics was performed by repeated measures two-way ANOVA with Bonferroni multiple comparisons test (*p < 0.05).

The effect of reduced hippocampal SK2 channel function on learning and memory was investigated in contextual fear conditioning, a hippocampus-dependent learning task. Mice were injected intrahippocampally (i.h.) or i.c.v. with either SK2 antisense ODNs, vehicle, control ODNs, or with the highly selective SK2 channel antagonist Lei-Dab^7^ 2 h and 0.5 h before the training session, respectively. The concentration of Lei-Dab^7^ was selected based on the finding that this concentration was shown to selectively block >99% of SK2 channel homotetramers without generating any central nervous system toxicity [10]. We never detected seizure-like behavior or changes in baseline motor activity during training in mice treated with SK2 antisense or Lei-Dab^7^. When re-exposed to the fear conditioning box 24 h after the training, animals injected with SK2 antisense ODN i.c.v. (two way repeated-measures ANOVA for test and treatment, main effect of treatment: F_3, 108_ = 33.1; *p* < 0.05) (Fig 3A), or injected i.h. (two way repeated-measures ANOVA, main effect of treatment: F_2, 69_ = 50.2; *p* < 0.05) (Fig 3B) showed reduced freezing responses compared to vehicle-treated animals (Bonferroni correction, *p* < 0.05; SK2 AS vs. Vehicle). Intrahippocampal injection of Lei-Dab^7^ resulted in similar reduction of contextual fear memory retention (two way repeated-measures ANOVA, main effect of treatment, F_1, 57_ = 86.1; *p* < 0.05) (Fig 3C). In order to exclude the possibility that the behavioral effects were actually mediated by inhibition of SK2 channel function in the cortex and not in the hippocampus, which might be the case if compounds diffused from the injection-site into cortical areas (i.c.), we injected Lei-Dab^7^ 0.5 h before the training into the cortex right above the hippocampal injection site. This treatment had no effect on contextual freezing measured 24 h later (Fig 3D). There was no significant difference between the groups in freezing level before the foot-shock presentation (pre-shock), suggesting that prior treatment did not induce unconditioned freezing response (Bonferroni correction; *p* > 0.05). Similarly, freezing in the post-shock period and 1 hour after conditioning was not different between animal groups (Bonferroni corrections; *p* > 0.05), indicating that blockade of SK2 channel function had no significant effect on short-term memory (Fig 3A–3C).

**Fig 4:**
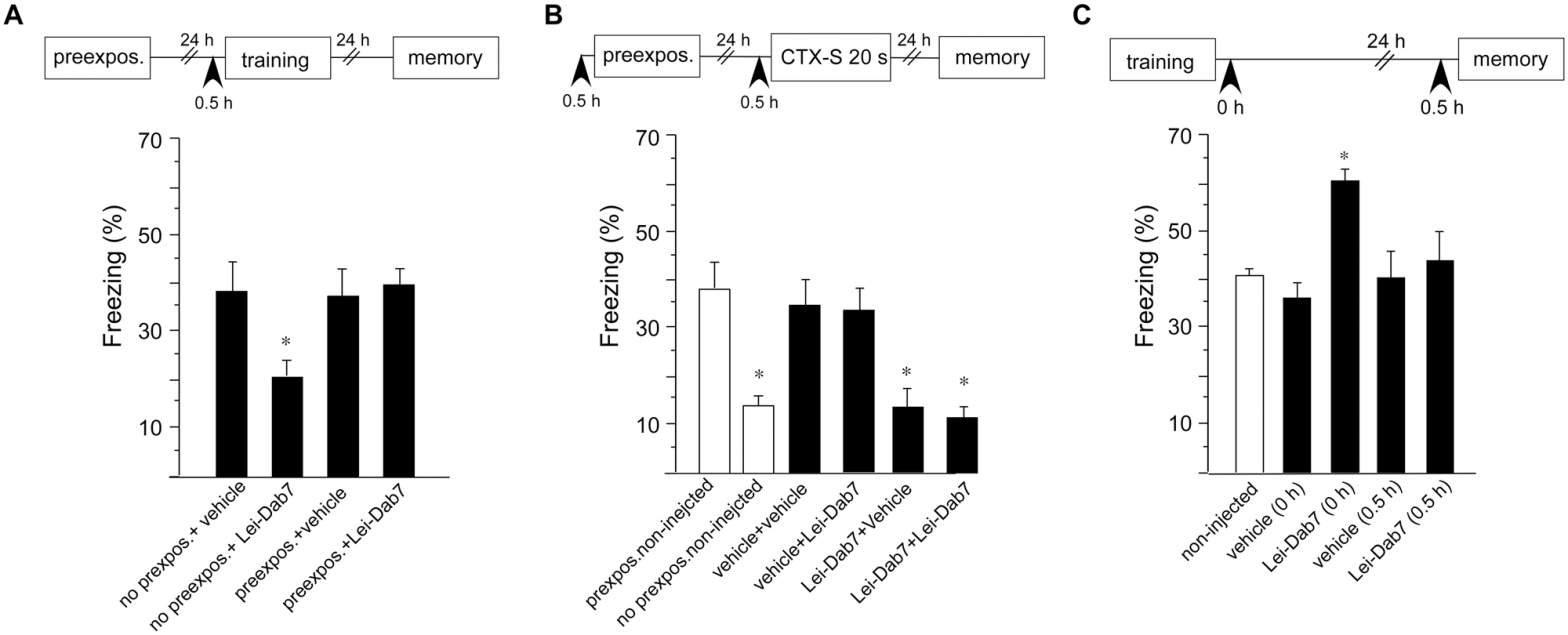
Pre-exposure of mice overcomes impaired contextual fear after inhibition of SK2 channel function. Foot-shock intensity was reduced from 0.7 mA to 0.5 mA. (A) Intrahippocampal injection of the SK2 antagonist Lei-Dab^7^ before training impaired freezing when compared to non-treated mice. The same treatment showed no impairment when animals were pre-exposed for 5 min to the conditioning context 24 h before the training. Vehicle-treated animals were not different from non-treated animals (n = 7/group). (B) The effects of SK2 blockade before pre-exposure to the conditioning context on subsequent contextual fear conditioning in which 20 sec placement to shock interval is employed (CTX-S 20s). Injection of Lei-Dab^7^ before 5 min pre-exposure to context eliminated facilitation of the acquisition of context conditioning at a 20 sec placement to shock interval. Injection of vehicle before pre-exposure or 20 sec placement to shock training phase did not influence contextual pre-exposure effect (n = 8–9/group). (C) Lei-Dab^7^ was injected at the indicated time points after the training and before the memory test. Statistical comparison was made versus non-injected animals (n = 7–9/group). Arrows in the schematic experimental diagram indicate time points of Lei-Dab^7^ injection. Freezing was measured in the memory test 24 h after training. Data presented are the mean ± SEM. Statistics was performed by two-way ANOVA with Bonferroni multiple comparisons test (*p < 0.05).

We then lowered the foot-shock intensity from 0.7 mA to 0.5 mA in order to enlarge the response window of the animal towards a potential increase in freezing behavior. This decision was based on the reported observation, that blockade of SK channels by apamin resulted in enhanced hippocampus-dependent learning [2]. Under these lowered foot-shock conditions, injection of Lei-Dab^7^ into the hippocampus of naïve animals 0.5 h before training (no prexposure Lei-Dab^7^ group) again resulted in reduced contextual fear when compared to non-treated mice (two-way ANOVA for exposure and treatment, main effect of treatment: F_1, 24_ = 8.7; *p* < 0.05) (Bonferroni correction; *p* < 0.05) (Fig 4A). One reason for impaired contextual fear can be the inability to form a contextual representation, which has to be associated with the foot-shock. To test this hypothesis, animals were pre-exposed for 5 min to the conditioning chamber 24 h prior to the actual training. In pre-exposed animals, the Lei-Dab^7^-induced impairment in contextual fear was not observed (two way ANOVA, main interaction effect (treatment x exposure), F_1, 24_ = 10.9; *p* < 0.05) (Bonferroni correction; *p* < 0.05; no-prexposed+ Lei-Dab^7^ vs. prexpos. + Lei-Dab^7^). Vehicle-treated animals were not different from non-treated animals (Fig 4A).

To further investigate the role SK2 channels in contextual representation formation, we employed context pre-exposure facilitation effect that selectively engages hippocampus-dependent conjunctive associations [15]. In the employed variant of pre-exposure to the context, 5 min context chamber pre-exposure is followed 24 h later by training using a 20 sec context chamber placement to shock interval. Such 5 min pre-exposure results in substantial conditioned freezing to the context in a subsequent test, relative to control animals that do not receive context pre-exposure. Animals treated i.h. with Lei-Dab^7^ 30 min before 5 min pre-exposure and vehicle before the 20 sec placement to shock interval training phase displayed contextual fear (Lei-Dab^7^ + vehicle group) comparable to that observed in mice that were not exposed to context pre-exposure (ANOVA, F_5, 44_ = 25.3; *p* < 0.05) (Bonferroni correction; *p* > 0.05), and reduced when compared to animals injected with vehicle before both pre-exposure and training (vehicle + vehicle group) (Bonferroni correction; *p* < 0.05). When animals were treated i.h. with Lei-Dab^7^ before the pre-exposure, injection of Lei-Dab^7^ before the 20 sec placement to shock interval training phase (Lei-Dab^7^ + Lei-Dab^7^ group) induced no further impairment of contextual fear when compared to the Lei-Dab^7^+vehicle group (Bonferroni correction; *p* > 0.05) (Fig 4B). Overall, these findings suggest that SK2 channels are critically involved in processing and retention of the contextual representation.

In order to determine the role of SK2 channels in the consolidation and retrieval of contextual fear memory, mice were i.h. injected with Lei-Dab^7^ 0 h post-training or 0.5 h before the retention test (Fig 4C). Inhibition of SK2 channels immediately after the training resulted in enhanced contextual fear memory (F_4, 35_ = 20.1; *p* < 0.05) (Bonferroni correction; *p* < 0.05 vs non-injected mice). On the other hand, inhibition of SK2 channels by Lei-Dab^7^ injection 0.5 h before the retention test showed no effect on memory performance (Fig 4C).

### Cleavage of the SK2 Channel in the C-terminus following Contextual Fear Conditioning

**Fig 5:**
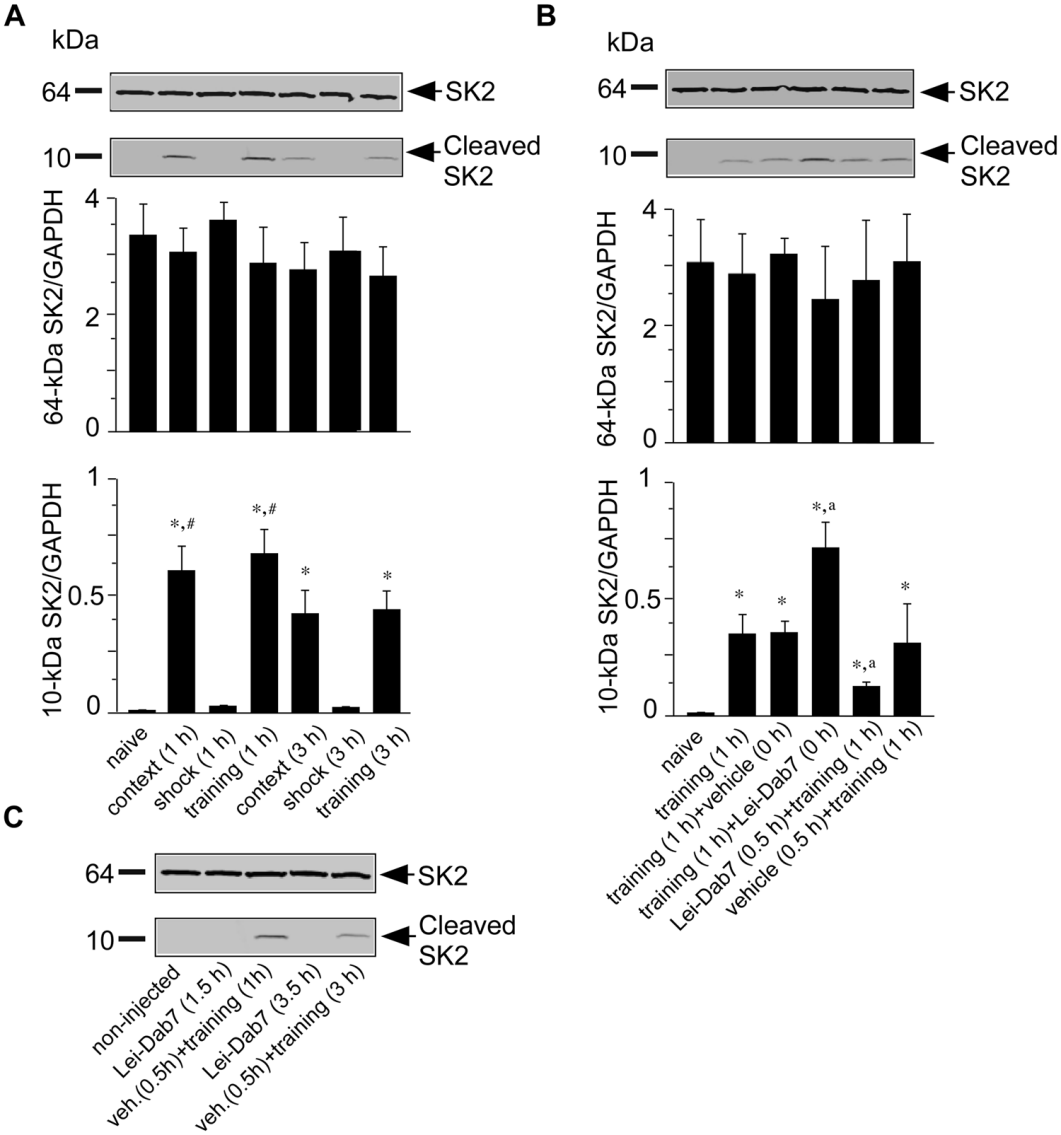
Cleavage of SK2 channel protein after contextual fear conditioning training. Western blot analysis of SK2 protein levels in (A) hippocampal tissue obtained from naïve mice or 1 h and 3 h after training (context+shock). In the context group, mice were exposed to the training context without receiving a foot-shock. In the shock group, mice received a foot-shock immediately after they were exposed to the training context and were removed immediately after the foot-shock. Experiment was performed twice. (B) Representative immunoblot of SK2 protein levels in hippocampal tissue from naïve, trained and trained vehicle- or Lei-Dab^7^-injected mice. Mice were injected either 0.5 h before or 0 h after training as indicated. Hippocampal tissue was removed 1 h after training. The SK2_(538–555)_ antibody recognized the 64-kDa SK2 protein and a 10-kDa SK2 C-terminal fragment (top). (C) SK2 protein levels in hippocampal tissue from naïve non-injected, Lei-Dab^7^-injected naïve, or vehicle-injected trained mice. Hippocampal tissue was removed 90 or 210 min after injection (1h and/or 3h after training). The number of individual samples per treatment was five. Data presented are the mean ± SEM. Statistically significant differences: *p < 0.05 versus naive, ^#^ *p* < 0.005 versus 3 hours context and conditioning groups, ^a^ *p*<0.05 versus training + vehicle.

To gain further insights into the mechanisms by which SK2 channels are involved in contextual fear memory consolidation, we performed Western immunoblots to analyze hippocampal SK2 protein levels in naïve mice and 1 h or 3 h after the training procedure with the help of an SK2 antibody raised against an epitope from the distal C-terminal domain [16]. We detected the expected 64-kDa protein band corresponding to full-length SK2 and a 10-kDa protein band 1 h and 3 h after fear conditioning and exposure to the context alone (ANOVA, F_6, 28_ = 10.3; *p* < 0.05) (Bonferroni correction; *p* < 0.05 vs. naïve mice). The SK2 10-kDa band intensity obtained with the 3 h samples was decreased when compared to the 10-kDa band intensity detected with the 1 h samples (Bonferroni corrections; ^#^ *p* < 0.05; 1 h training/context vs. 3 h training/context groups). This SK2 10-kDa band was absent in hippocampal lysates from naïve mice as well as in the shock control group (Fig 5A).

Next, we asked if Lei-Dab^7^ injection might affect the observed SK2 C-terminal cleavage following training. We found that the SK2 10-kDa band visible one hour after training was significantly enhanced by prior i.h. injection of Lei-Dab^7^ immediately after training (ANOVA, F_5, 24_ = 11.5; *p* < 0.05) (Bonferroni correction; *p < 0.05 vs naïve, ^a^ *p* < 0.05 vs. training + vehicle group). Band intensity for the full-length 64-kDa SK2 protein was unaltered (Fig 5B). Interestingly, the SK2 10-kDa band observed after training was significantly reduced when animals were pre-injected with Lei-Dab^7^ 0.5 h before training (Bonferroni correction; ^a^ *p* < 0.05 vs. training + vehicle group) (Fig 5B). Pre-injection with vehicle had no effect on the SK2 10-kDa band intensity. In order to determine whether Lei-Dab^7^ injection might also induce a cleavage of the SK2 channel protein at the C-terminus without training, we injected naïve mice i.h. with Lei-Dab^7^ and sacrificed the animals 1.5 or 3.5 h later (Fig 5C), corresponding to the time points when Lei-Dab^7^- dependent modulation of a training-induced SK2 C-terminal cleavage was observed (Fig 5B). We only detected the full-length 64-kDa SK2 protein-band without any SK2 10-kDa band after Lei-Dab^7^ injection. This indicated that Lei-Dab^7^ injection itself induced no detectable cleavage of the SK2 channel protein (Fig 5C) (Bonferroni corrections; *p* > 0.05).

### SK2 Leucine Zipper Domain and Contextual Fear Conditioning

**Fig 6:**
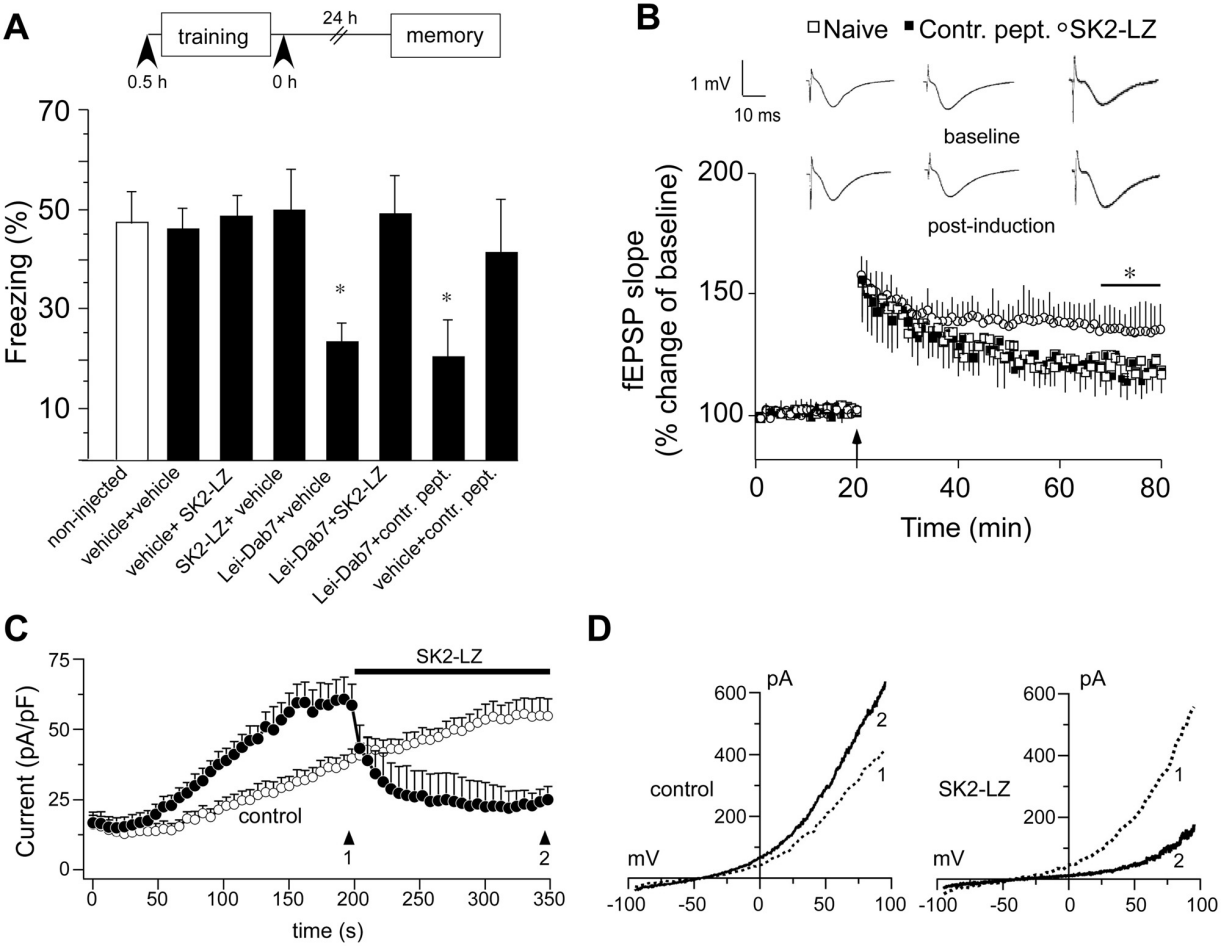
SK2 leucine zipper (SK2-LZ) domain peptide enhances fear conditioning and TBS-LTP by inhibiting SK2 current. (A) Mice intrahippocampally (i.h.) injected at 30 before or immediately after training with either the SK2-LZ peptide _(488–526)_, a random sequenced control peptide or vehicle showed no difference in freezing when compared to non-injected animals. Injection of the selective SK2 antagonist Lei-Dab^7^ 0.5 h before training impaired freezing when compared to non-injected animals. This impairment was rescued if mice were injected with the SK2-LZ peptide _(488–526)_ immediately after training. I.h. injection of SK2-LZ peptide _(488–526)_ alone, either 30 min before training or immediately following training did not affect contextual fear. Freezing was measured in the memory test 24 h after training. n = 7–10 mice/group. Statistics were performed by one-way ANOVA with Bonferroni multiple comparisons test (*p < 0.05). (B) TBS-LTP elicited in slices from mice that were pre-treated with SK2-LZ peptide _(488–526)_ was significantly enhanced when compared to LTP induced in slices from naive mice. There was no statistical difference between control peptide-injected mice and naïve mice. Statistics were performed by two-way ANOVA with Bonferroni multiple comparisons test (*p < 0.05). Insets: Responses shown are fEPSPs recorded during baseline (upper row) and 55–60 min (bottom row) after the induction of LTP (post-induction). Traces are averages of five consecutive responses. (C) Average whole-cell currents were recorded in Jurkat cells endogenously expressing SK2 channels. Voltage ramps were from -100 mV to 100 mV for 100 ms delivered at 2 s intervals. SK2 current was measured at 80 mV and normalized to cell size (in pF) to show current density over time. After 200 seconds external solutions containing 100 μM SK2-LZ peptide (filled circles, n = 6) was applied to the cells for 150 seconds or no external solution application (control; open circles, n = 4). Arrows at 196 s and 348 s indicate the time points at which the current-voltage (I-V) curves in D were obtained (D) Representative I-V curves for endogenous SK2 in Jurkat cells taken after 196 s, right before application (dotted line) and at the end of the experiment at 348 s (solid line) in unexposed control cells (left panel) or when exposed to 100 μM SK2-LZ peptide. Data presented are the mean ± SEM.

In close proximity to the binding site of the C-terminal anti-SK2_(538–555)_ antibody lies a recently described leucine zipper (LZ) domain (488–526) [17]. Increasing evidence suggests that LZ domains play an important role in both the assembly of ion channel signaling complexes as well as ion channel assembly *per se* [18,19]. To investigate whether the SK2-LZ motif plays a functional role as part of the cleaved SK2 C-terminal fragment we synthesized the SK2-LZ peptide and determined its effects in contextual fear conditioning. Intrahippocampal injection of the synthesized SK2-LZ peptide 30 min before or immediately after the training procedure had no significant effect on contextual fear when compared to non-injected mice (Fig 6A). However, injection of the SK2-LZ peptide immediately after training could overcome the Lei-Dab^7^–induced impairment (injection 30 min before training) in contextual fear so that freezing behavior was indistinguishable from that of non-injected mice (ANOVA, F_7, 62_ = 24.9; *p* < 0.05) (Bonferroni corrections; *p* < 0.05 vs. non-injected mice). This rescue of the contextual fear memory impairment was not observed when a random-sequence control peptide was injected at time zero after training (Fig 6A).

We then tested the potential impact of SK2-LZ and control peptide i.c.v.-injected 6 h before decapitation on LTP recordings in the CA1 region of the hippocampus. In mice injected with control peptide theta-burst stimulation induced a weak, decaying form of fEPSP-LTP (121 ± 6% of baseline; 8 slices, 6 mice), which was not significantly different from that observed in naïve animals (117 ± 2% of baseline; 10 slices, 7 mice) (Fig 6B). In hippocampal slices prepared from animals injected with SK2-LZ peptide TBS resulted in significantly enhanced fEPSP-LTP when compared to naïve mice (140 ± 9% of baseline; 10 slices, 8 mice) (F_2, 25_ = 9.7; *p* < 0.05; ANOVA with repeated-measures, last 10 min) (Bonferroni corrections; *p* < 0.05 vs. Naive) (Fig 6B).

Taking into account that the application of the SK2-LZ peptide enhanced TBS-LTP, we next tested if the SK2-LZ peptide would directly inhibit SK2 channel function. We addressed this question with the help of whole-cell patch-clamp experiments performed with Jurkat T cells, which endogenously express SK2 channels [20]. Intracellular perfusion of cells with pipette solutions in which the Ca^2+^ concentration was buffered to 1.9 μM induced the development of SK2 currents at 80 mV (Fig 6C). In cells exposed to external solution containing 100 μM SK2-LZ peptide the outward current was strongly inhibited (Fig 6C). These effects are also evident in the current-voltage (I/V) relationships obtained from exemplary individual cells assessed through membrane currents in response to voltage ramps, demonstrating the inhibitory effect of SK2-LZ peptide (Fig 6D, right panel) and lack thereof in unexposed control cells (Fig 6D, left panel).

## Discussion

The main finding of the present study is a dual role of SK2 channels in the dorsal hippocampus to regulate synaptic plasticity and the formation of contextual fear. We report that SK2 channels act as ion channels and can be cleaved to produce C-terminal fragments during contextual fear conditioning. Initially, we showed that both, reduction of hippocampal SK2 channel expression by SK2 antisense treatment and SK2 channel blockade by the selective SK2 channel antagonist Lei-Dab^7^ before training impaired contextual fear memory in both cases by approximately 45%. This finding might seem surprising considering that ODN probes against SK2 reduced SK2 protein by approximately 50% whereas the Lei-Dab^7^-treatment was expected to block about 99% of SK2 channels. The fact that Lei-Dab^7^-treatment was not more effective in impairing contextual fear than the ODN treatment suggests that electrical activity of the SK2 channel complex cannot be the only signal necessary for contextual fear memory formation. If we assume that the amount of cleaved SK2 C-terminal peptide parallels the intensity of contextual fear memory, it is interesting to note that Lei-Dab^7^-treatment before training reduced the level of the 10 kDa SK2 C-terminal fragment by approximately 50% compared to trained, non-injected mice. A similar reduction in the amount of the SK2 C-terminal peptide by approximately 50% could be expected after SK2 ODN treatment and might explain similar contextual freezing levels seen after both treatments.

Electrophysiological slice recordings from animals treated with SK2 antisense probes revealed enhanced baseline synaptic transmission and facilitated LTP in the hippocampal CA1 area. These findings are consistent with previous studies, which reported that SK2 channels are mainly found at the postsynaptic site in the CA1, CA2 and CA3 pyramidal cell layers of the hippocampus [16]. In one study, it was shown that Lei-Dab^7^ increases the facilitation of burst responses that occur within a theta stimulation train relative to controls. These changes in burst-response characteristics generated by blockade of SK2 with Lei-Dab^7^ are accompanied by an increase in TBS-induced fEPSP-LTP [21]. Taking into consideration that in our experiments SK2 channel downregulation by antisense treatment was also associated with enhanced fEPSP-LTP, it is assumed that reduced SK2 channel function is crucial to facilitate synaptic plasticity within the CA1 hippocampal network. This assumption is in accordance with recordings from hippocampi of transgenic mice overexpressing SK2 channels, which show reduced LTP after high-frequency stimulation [7]. However, the neuronal function of SK2 channels depends highly on where the channels are overexpressed within hippocampal subfields. Overexpression of SK2 channels in dentate gyrus granule cells has been shown to result in a steeper increase of the fEPSP slope with increasing stimulation intensity than in the control group [8]. In behavioral experiments, SK2 overexpression in dentate gyrus granule cells had no effect on memory acquisition in the Morris water maze but a posttraining memory deficit was detectable [22]. Since most of our injections were restricted to the hippocampal CA1 area, it is very difficult to compare our findings with previous reports indicating that blocking SK channels accelerates hippocampal-dependent spatial memory encoding [2]. This group was using the non-selective SK channel blocker apamin in their study, which was given systemically and not region-restricted.

Contextual fear is mediated by a neural circuit, in which the hippocampal formation is thought to play an important role by forming a representation of the training environment [23–27]. It might be conceivable that pre-training injection of Lei-Dab^7^ impaired contextual fear by antagonizing the acquisition of the mouse’s contextual representation. We found that the impairment of contextual fear in mice pre-injected with the SK2 channel antagonist before the training was overcome in animals pre-exposed to the conditioning context. The fact that the beneficial effect of context pre-exposure vanished in the presence of the SK2 channel blocker during contextual pre-exposure paradigm indicated that SK2 channels in the dorsal hippocampus are critical to acquire a representation of the context. This finding is in agreement with the observation that contextual memory impairment of transgenic mice overexpressing SK2 channels is eliminated if mice are permitted longer pre-exposure to the conditioning chamber [8]. It is believed that the hippocampus contributes to memory by automatically encoding and storing conjunctive representations of co-occurring features that define particular environments [25]. For example, it is proposed that the hippocampus is connected to exploration and that much of what it encodes is the result of novelty-directed behaviors [27]. Our results indicate that SK2 channels in the hippocampus are, similar to NMDA receptors [28], critically involved in the acquisition of memory representations automatically acquired as a consequence of exploring a novel environment. Functionally, SK channels modulate NMDA receptors activity via a negative feedback loop where Ca^2+^ entry via NMDA receptors activates SK channels and potassium flux, which hyperpolarizes the membrane resulting in NMDA receptor inactivation [29]. Concordantly, another study shows that enhanced SK2 channel activation mediates negative feedback on NMDA receptors and causes impairment of CA1 fEPSP-LTP, and that inhibition of SK2 channel activity restores NMDA receptor function and CA1 fEPSP-LTP [30]. Whether the blockade of SK2 channels enhances CA1 fEPSP-LTP by enhancing NMDAR-dependent Ca^2+^ signals only, while for the memory enhancement both the blockade and the cleavage of the C-terminal SK2 fragment, are necessary as observed following post-training Lei-Dab^7^ application remains to be tested. Although highly speculative, in that case, pre-training application of Lei-Dab^7^ might enhance NMDA receptor function but decrease subsequent cleavage of the C-terminal SK2 fragment leading to impaired fear conditioning. Thus, SK2-mediated modulation of NMDA receptor activity seems to be a necessary but, on its own, an insufficient condition for memory formation. Obviously it is also critical, at which point during fear conditioning the SK2 fragment is freely available in the cell in order to allow, together with enhanced NMDA receptor activity, the memory to be formed. It has long been known that memory consolidation is a time-dependent process [31]. In hippocampus, double-label immunogold electron microscopy revealed close coclustering of SK2 channels and mGlu_5_ receptors in dendritic spines of CA1 pyramidal neurons. Activation of mGlu_5_ receptors mobilizes intracellular Ca^2+^, which gates the SK2 channel to decrease excitability [32]. On the other hand, SK2 channels potentiate mGlu_5_ receptor functioning. It is conceivable that the C-terminal SK2 fragment inhibits SK2 channel function, which in turn might inhibit mGlu5 with the consequence of elevated neuronal activity.

We were not able to determine whether the cleaved 10-kDa C-terminal fragment might also contain a part of calmodulin (CaM)-binding domain (CaMBD, 407–483), which lies immediately following the sixth transmembrane domain and in close proximity of leucine zipper (LZ) domain (488–526). If so, following the cleavage, the channel might lose functionality by losing the ability to interact with calmodulin, which serves as a Ca^2+^ sensor and is mandatory for gating [33] and subsequently for dendritic integration [32].

Our electrophysiological experiments in cell culture demonstrated that the cleaved C-terminal SK2 fragment directly modulates the functional characteristics of SK2 channels. In addition to modulating channel function, the cleaved C-terminal SK2 fragment might also exert its physiological function via transcriptional regulation. It was recently shown that a C-terminal fragment of CaV1.2, anL-type voltage-gated calcium channel (LTC), translocates to the nucleus and regulates transcription [34]. The proteolytically processed C-terminal domain is also thought to bind to truncated channels, where it exerts an inhibitory effect on channel function [19]. In the case of SK2, we found that a putative leucine-zipper motif in the C-terminal amino acid sequence played a functional role in contextual fear memory formation and synaptic plasticity. The actual mechanism, and the question whether other motifs in the cleaved SK2 C-terminal fragment play an important functional role during memory consolidation and long-term synaptic plasticity remain to be elucidated.

In conclusion, we have demonstrated that hippocampal SK2 channels play a critical role during encoding and consolidation of contextual fear. Fear conditioning favours C-terminal cleavage of SK2 channels where the C-terminal fragment has the ability to block SK2 channels and thus enhance synaptic potentiation. The highly time-dependent involvement of SK2 channels in fear memory consolidation seems to consist of an entirely novel and unsuspected function, which is initiated by C-terminal cleavage of SK2 channel proteins.
